# Sensitivity to negative and positive feedback as a stable and enduring behavioural trait in rats

**DOI:** 10.1007/s00213-019-05333-w

**Published:** 2019-08-03

**Authors:** Karolina Noworyta-Sokolowska, Anna Kozub, Judyta Jablonska, Jan Rodriguez Parkitna, Robert Drozd, Rafal Rygula

**Affiliations:** 1 Affective Cognitive Neuroscience Laboratory, Department of Pharmacology, Maj Institute of Pharmacology Polish Academy of Sciences, 12 Smetna Street, 31-343 Krakow, Poland; 2 Department of Molecular Neuropharmacology, Maj Institute of Pharmacology Polish Academy of Sciences, 12 Smetna Street, 31-343 Krakow, Poland

**Keywords:** Feedback sensitivity, Animal model, Behavioural trait, Cognitive bias, Reinforcement learning

## Abstract

**Rationale:**

According to psychological theories, cognitive distortions play a pivotal role in the aetiology and recurrence of mood disorders. Although clinical evidence for the coexistence of depression and altered sensitivity to performance feedback is relatively coherent, we still do not know whether increased or decreased sensitivity to positive or negative feedback is associated with ‘pro-depressive’ profile in healthy subjects.

**Objective:**

Our research has been designed to answer this question, and here, we present the first steps in that direction.

**Methods:**

Using a rat version of the probabilistic reversal-learning (PRL) paradigm, we evaluated how sensitivity to negative and positive feedback influences other cognitive processes associated with mood disorders, such as motivation in the progressive ratio schedule of reinforcement (PRSR) paradigm, hedonic status in the sucrose preference (SP) test, locomotor and exploratory activity in the open field (OF) test, and anxiety in the light/dark box (LDB) test.

**Results:**

The results of our study demonstrated for the first time that in rodents, sensitivity to negative and positive feedback could be considered a stable and enduring behavioural trait. Importantly, we also showed that these traits are independent of each other and that trait sensitivity to positive feedback is associated with cognitive flexibility in the PRL test. The computational modelling results also revealed that in animals classified as sensitive to positive feedback, the α learning rates for both positive and negative reward prediction errors were higher than those in animals classified as insensitive. We observed no statistically significant interactions between sensitivity to negative or positive feedback and the parameters measured in the PRSR, SP, OF or LDB tests.

**Conclusions:**

Further studies using animal models of depression based on chronic stress should reveal whether sensitivity to feedback is a latent trait that when interacts with stressful life events, could produce correlates of depressive symptoms in rats.

## Introduction

Several cognitive models have proposed that cognitive distortions and affective biases play a crucial role in the aetiology and recurrence of affective disorders (Beck 1967, 1987). According to the model posed by Beck, inappropriate cognitive patterns may remain inactive or dormant, but when activated by stress, they can lead to depression (Beck 1987). Mentioned distortions have been observed in clinical trials (Beats et al. 1996; Brittlebank et al. 1993; Murphy et al. 2003) and include biased attention, biased interpretation and biased memory, as well as aberrant sensitivity to performance feedback (Disner et al. 2011; Gotlib and Joormann 2010). The last-mentioned phenomenon manifests itself as an overreaction to negative events (catastrophic reaction to perceived failure) or as underestimation of the value of positive situations (Beats et al. 1996; Elliott et al. 1997; Murphy et al. 2003; Taylor Tavares et al. 2008). Despite relatively coherent clinical evidence of the co-occurrence of altered sensitivity to feedback and depressed mood (Beats et al. 1996; Elliott et al. 1997; Murphy et al. 2003; Taylor Tavares et al. 2008), it is still unclear whether this bias co-occurs with ‘pro-depressive’ profile in healthy subjects.

Recent (Bari et al. 2010) implementation of the pre-clinical version of the probabilistic reversal learning (PRL), a paradigm that allows for measurement of altered sensitivity to feedback in humans (Maia and Frank 2011), enabled investigation of this question in animal models (for review, see Rygula et al. (2018)). In the PRL task dedicated to rodents, animals are tested in operant conditioning boxes, where they learn to discriminate between 2 levers. Pressing one of them is associated with a high probability of the rewarding outcome and low probability of punishment and vice versa for the other lever. This rule intermittently reverses so that the lever that was usually rewarded becomes usually punished (or non-rewarded) and the usually punished one becomes usually rewarded. For successful completion of the task, rats must ignore infrequent misleading negative feedback arising from the probabilistic nature of the discrimination (Bari et al. 2010; Dalton et al. 2014; Dalton et al. 2016; Ineichen et al. 2012; Rychlik et al. 2017; Rygula and Popik 2016).

Studies using the PRL task in animals have demonstrated that feedback sensitivity is modulated by serotoninergic, dopaminergic and glutamatergic neurotransmission in rodents, which is similar to that in humans (Bari et al. 2010; Grospe et al. 2018; Rychlik et al. 2017). Other studies have revealed that alterations in feedback sensitivity are associated with the activity within brain regions that are commonly linked with mood disorders, such as prefrontal and orbitofrontal cortices (Dalton et al. 2016) and the ventral striatum (Dalton et al. 2014). Despite the growing amount of pre-clinical evidence, there is a paucity of information about the causal relationship between altered sensitivity to feedback and behavioural correlates of sensitivity to depression.

In our study, using a pre-clinical version of the PRL task, we investigated whether low/high sensitivity to negative and positive feedback could be associated with a ‘pro-depressive’ behavioural profile in rats. For this purpose, using a series of the PRL tests, we initially classified each individual animal as sensitive or insensitive to negative feedback and as sensitive or insensitive to positive feedback. The rats classified in this way were subsequently tested in a battery of behavioural tests aimed at measuring the motivational, hedonic and affective (anxiety) correlates of depressive symptoms in rodents. Appetitive motivation was assessed in the progressive ratio schedule of reinforcement (PRSR) paradigm, hedonic status was evaluated in the sucrose preference (SP) test, locomotor and exploratory activities were measured in the open field (OF) test, and finally, the level of anxiety was measured in the light/dark box (LDB) test. Lastly, three different reinforcement-learning models were fitted (and tested along with the ‘random choice’ model) to the behavioural data to further assess potential differences between the feedback-sensitive and feedback-insensitive animals using computational modelling. The simplest, ‘basic’ model assumed a single learning rate for both positive and negative choice outcomes. The second model, ‘dual’, introduced separate learning rates, while the third, ‘fictitious’, assumed a single learning rate but added an update of expected value for the non-selected option (den Ouden et al. 2013; Glascher et al. 2009; Rescorla and Wagner 1972).

## Materials and methods

### Ethics statement

All experiments were conducted in accordance with the European Union guidelines for the care and use of laboratory animals (2010/63/EU). Experimental protocols were reviewed and approved by the 2nd Local Institutional Animal Care and Use Committee, Institute of Pharmacology Polish Academy of Sciences in Krakow. The authors attest that all efforts were made to minimize the number of animals used and their suffering.

### Subjects and housing

In the present study, we used 40 male Sprague–Dawley rats (Charles River, Germany) weighing 175–200 g upon arrival. Rats were kept in groups (4 animals/cage) under controlled temperature (21 ± 1 °C) and humidity (40–50%) under a 12/12 h light/dark cycle (lights on at 07:00). During the entire experiment, rats were mildly food restricted to 85% of their free-feeding weight (according to normal growth curve recommended by the laboratory rodent supplier—Charles River Research Models and Services Catalogue) by providing 15–20 g of food pellets per rat per day (standard laboratory chow). The food restriction began 1 week prior to behavioural training. Water was available *at libitum*. All of the conducted behavioural procedures and tests were performed during the light phase of the light/dark cycle.

### Apparatus

The behavioural tests were performed in 16 computer-controlled operant conditioning boxes (Med Associates; St Albans, VT, USA). Boxes were equipped with a light, fan, speaker, a food dispenser set to deliver a sucrose pellet (Dustless Precision Pellets, 45 mg; Bio-Serv, New Jersey, USA), and two retractable levers were located on opposite sides of the feeder. All applied behavioural protocols were programmed in Med State notation code (Med Associates) and analysed with a custom written R program. The experimental procedure for the PRL task used in this study was a modified version of the procedures used and described previously by Bari et al. (2010) and Dalton et al. (2014), and the procedure has been described in detail elsewhere (Drozd et al. 2019; Rychlik et al. 2017).

### Measuring feedback sensitivity using PRL test

#### Initial training

In the first stage, one of the levers (left/right counterbalanced between stages/animals) was extended, and every press on this lever was rewarded with sugar pellet delivery (fixed ratio schedule of reinforcement 1:1), after which the lever retracted for 3 s (inter-trial interval (ITI)) before the next trial commenced. No response within 10 s from lever presentation was marked as an omission, and a criterion of less than 20% omissions had to be met before progressing to the second stage of the training. Training sessions lasted 30 min, and there was no pre-set limit of trials.

The second stage of training consisted of random presentations of either the left or right lever, each of which had to be pressed at least 30 times in 30 min. To avoid side bias during the PRL task, animals had to respond with similar frequency on both levers. This was achieved by training the rats to a criterion of less than 7.5% omissions on each lever (i.e., less than 15% total omissions but equally distributed between the levers) for 3 consecutive training days. After attaining this criterion, the animals were ready to be tested in the PRL procedure.

### PRL training and testing

The PRL procedure used in our study was previously described (Drozd et al. 2019). Each PRL training session consisted of 200 trials, and each trial lasted for a maximum of 22 s. The start of a trial was signalled by the house light, which remained on until the end of the trial. Two seconds after the trial had started, both levers were presented, and one of them was randomly assigned as the ‘correct’ lever, which delivered a reward 80% of the times it was pressed. A press on the other lever—the ‘incorrect’ lever—would result in a rewarding outcome only 20% of the times it was pressed. No response in 10 s triggered the ITI and was counted as an omission. The same ITI directly followed a punishing outcome, i.e. no reward on 20% of the ‘correct’ and 80% of the ‘incorrect’ lever presses. After every 8 consecutive ‘correct’ lever presses (regardless of the outcome), the criterion for the reversal of the outcome probabilities was reached. The previously ‘correct’ lever now became ‘incorrect’ and vice versa. This pattern was followed until the end of the session.

This training phase was repeated daily until the individual animals achieved sufficient performance levels. The criteria to be met were a minimum of 3 reversals completed during 3 consecutive training sessions, with less than 15% omissions per session.

### Parameters measured in the PRL test

The animals’ decisions were tracked on a trial-by-trial basis to monitor their sensitivity to positive and negative feedback. To evaluate the ability of animals to ignore infrequent and misleading negative feedback, punished (non-rewarded) outcomes on the ‘correct’ lever, after which an animal decided to switch levers (probabilistic lose-shifts) were scored and expressed as a ratio of all punished (unrewarded) outcomes on that lever. To assess sensitivity to positive feedback, all rewarded outcomes (true and misleading) followed by a decision to stay with the lever that delivered them (win-stays) were counted jointly for the ‘correct’ and ‘incorrect’ levers and expressed as a ratio of all rewarded outcomes on that lever. This way of analysing sensitivity to positive feedback followed the method described by Bari et al. (2010) and was dictated by the fact that the win-stay behaviours following misleading rewards on the incorrect lever were too rare to undergo any robust analysis.

The number of reversals completed during the test was used to assess cognitive flexibility, which relies on the ability to both suppress previously rewarded action and engage in previously unrewarded actions (Nilsson et al. 2015).

### Measuring appetitive motivation using the PRSR

The PRSR tests were conducted in the same operant conditioning boxes that were used for the PRL paradigm and have been described elsewhere (Rygula et al. 2015). In brief, at the beginning of the PRSR test session, both levers were extended into the operant chamber. Pressing on one of the levers was reinforced with sugar pellet delivery, and the number of responses required to produce the next reward increased progressively with each successive reward obtained. The steps of the exponential progression used in our study were the same as those previously developed by Roberts and Bennett (1993) and previously used by Rygula et al. (2015) and were based on the following equation: response ratio = (5eX(0.2 × reward number)) − 5, rounded to the nearest integer. Thus, the values of the steps were 1, 2, 4, 6, 9, 12, 15, 20, 25, 32, 40, 50, 62, 77, 95, 118, 145, 178, 219, 268, 328, 402, 492, 603, etc. After each reward delivery, both levers retracted for a 10 s ITI. Test session lasted 30 min. Responding on the non-rewarded lever had no consequences. Each animal was only tested once.

### Measuring hedonic status using SP test

**Fig. 1:**
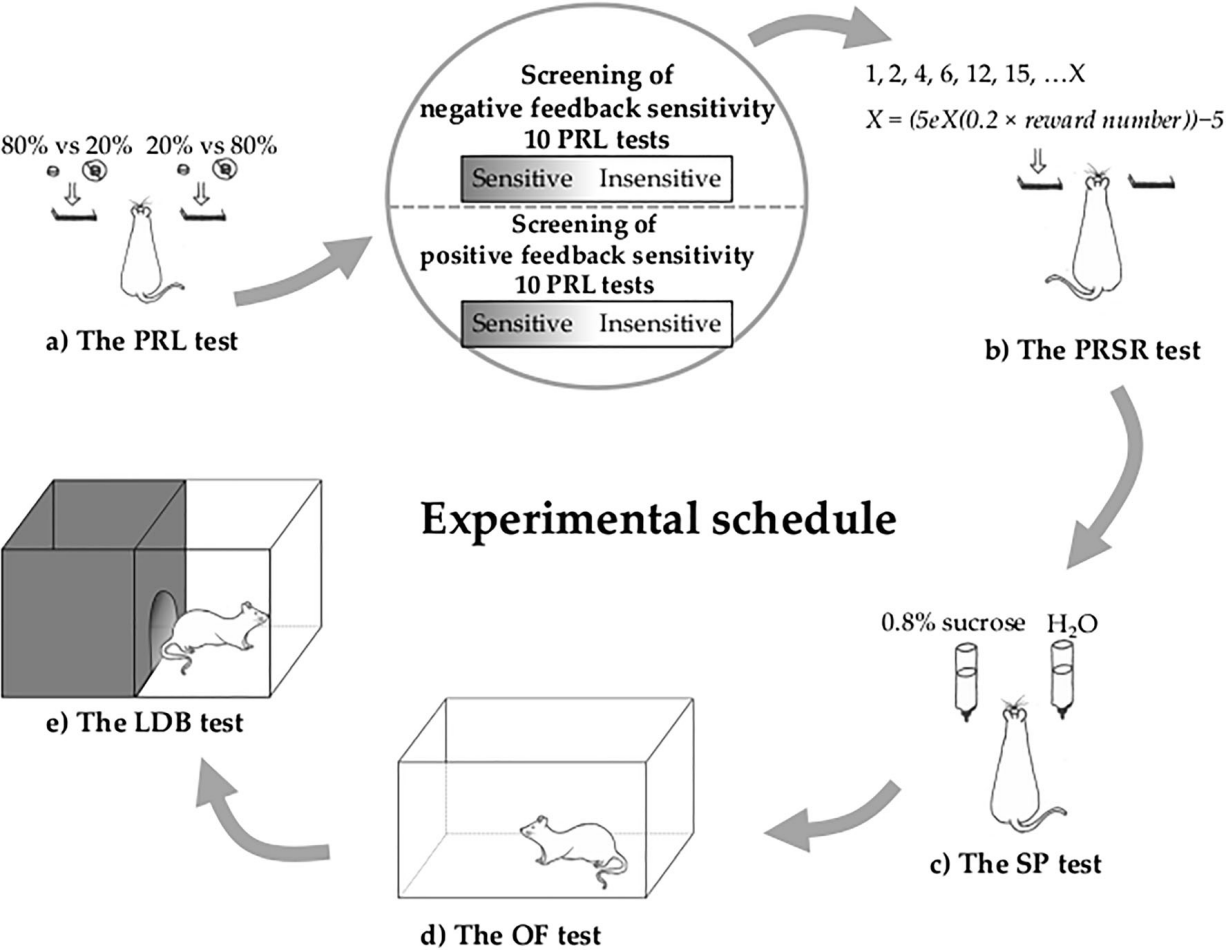
Experimental schedule. Initially, (a) using a series of PRL tests, we classified individual animals as sensitive or insensitive to negative feedback and sensitive or insensitive to positive feedback. The rats classified in this way were subsequently tested in a battery of behavioural tests aimed at measuring the motivational, hedonic and affective (anxiety) correlates of depressive symptoms in rodents (b, c, d and e). Appetitive motivation (b) was assessed in the PRSR paradigm, hedonic status (c) was evaluated in the SP test, locomotor and exploratory activities (d) were measured in the OF test, and finally, the level of anxiety (e) was measured in the LDB test

To assess the hedonic status in rats that were ‘insensitive’ and ‘sensitive’ to negative/positive feedback, the animals were subjected to SP tests (Fig. 1c). A decreased preference for the sweet sucrose solution has been previously postulated to be an indicator of anhedonia in rodents (Papp et al. 1991; Willner et al. 1992), and the applied test has been described elsewhere (Rygula et al. 2013). In brief, during the SP test, the rats were separated into single cages and were offered a voluntary choice between two bottles for 1 h, where one bottle contained a 0.8% (*w*/*v*) sucrose solution and the other bottle contained tap water. To prevent potential effects of side preference in drinking, the position of the bottles was switched after 0.5 h. The consumption of water and sucrose solution was measured by weighing the bottles. The preference for sucrose was calculated from the amount of sucrose solution consumed and is expressed as a percentage of the total amount of liquid that was consumed.

### Measuring locomotor and exploratory activity using the OF test and anxiety in the LDB test

#### Apparatus

The OF (Fig. 1d) and LDB (Fig. 1e) tests were performed in 4 computer-controlled Seamless Open Field Arenas (Med Associates; St Albans, Vermont, USA) for rats (43.38 cm L × 43.38 cm W × 30.28 cm H) that have 16 infrared emitters and photodetectors on each side of the box. Additionally, on two sides, there are emitters and detectors in two rows enabling measurement of rearings. For the LDB test, a dark insert was used, which divided the chamber into two equally sized compartments—a light compartment and a dark compartment with a hole between that allowed rats to move freely between the compartments. The data were collected using Med State software (Activity monitor, Med Associates).

#### Measures of interest

Before both tests, the rats were habituated to the experimental room for 30 min. Activity in the OF was measured for 5 min, while that in the LDB was measured for 15 min. The OF test started by placing a rat in the centre of the OF arena. The LDB test started by placing a rat in the centre of the dark zone. An additional light source (220 lm) was placed above the test chamber to make it more anxiogenic for a rat. Movement of rats was detected by beam breaks, and the whole system was computerized, which enabled reliable measurement of several different parameters, such as distance travelled, number and duration of rearings (in the OF test), latency to leave the safe/dark zone, and proportion of time spent in the anxiogenic/light zone (in the LDB test).

### Computational modelling

Four reinforcement-learning models (a—‘basic’, b—‘dual’, c—‘fictitious’, and d—‘random choice’) were fitted to trial-by-trial choice data from a total of 10 PRL screening tests. Optimal parameters were found using the Nelder and Mead simplex method (Nelder and Mead 1965) implemented in the R optim function repeated over 10 random starting points. Permitted value ranges were (0, 1) in the case of α and (0, 50) for β. The optimal parameters for each model were selected based on the lowest sum of the negative logarithm of likelihood (nll). The expected value of both options (reward vs. punishment (non-reward)) at the beginning of the given test (initial Q values) was set to 0.5.

The first fitted model, ‘basic’, assumed that animals learned according to prediction error (Rescorla and Wagner 1972). The ‘dual’ model assumed that animals learned with different rates when the prediction error was positive or negative (den Ouden et al. 2013). The ‘fictitious’ model assumed that rats learned that pressing one of the levers resulted in a high reward probability and influenced reward prediction error for both chosen and unchosen options. Thus, after choosing one option, the expected rewards for both options (‘correct’ and ‘incorrect’ lever) were updated in opposite directions (Glascher et al. 2009). The ‘random choice’ model assumed that rats pressed the levers in a random order.

In the ‘basic’ model, the expected value (*Q*_*t* + 1_) was updated according to prediction error (*R* – *Q*). Expected values were updated based on current information with learning rate α as follows:

$$ {Q}_{t+1}={Q}_t+\alpha \left({R}_t-{Q}_t\right)\kern4em \mathrm{where}\ R\in \left\{0,1\right\}\ \mathrm{and}\ \alpha \in \left\{0,1\right\} $$

In the ‘dual’ model, the expected value (*Q*_*t* + 1_) was updated according to prediction error (*R* – *Q*_*t*_). Expected values were updated based on current information with learning rate α that was different when the reward prediction error was positive and negative as follows:

$$ {Q}_{t+1}=\left\{\begin{array}{c}{Q}_t+{\alpha}_{+}\left({R}_t-{Q}_t\right)\kern2em \mathrm{if}\ R=1\\ {}{Q}_t+{\alpha}_{-}\left({R}_t-{Q}_t\right)\kern2em \mathrm{if}\ R=0\end{array}\right. $$

In the ‘fictitious’ model, the expected value of chosen (*Q*_*t* + 1,*c*_) and not chosen option (*Q*_*t* + 1,*nc*_) was updated according to prediction error. Expected values were updated based on current information with learning rate α depending on reward prediction error and varying between options as follows:

$$ {Q}_{t+1,c}={Q}_{t,c}+\alpha \left({R}_t-{Q}_{t,c}\right) $$

$$ {Q}_{t+1, nc}={Q}_{t, nc}-\alpha \left({R}_t-{Q}_{t,c}\right) $$

Choice probabilities were calculated using the softmax function as follows:

$$ p(a)=\frac{e^{{\beta Q}_{t,a}}}{\Sigma_{i=1}^n{e}^{{\beta Q}_{t,i}}} $$

The inverse temperature free parameter β indicates the degree of exploration and exploitation.

The models were compared using the Akaike information criterion (AIC, (Akaike 1981)) as follows:

$$ AIC=2 nll+2k $$

where $nll=\sum \limits_{i=1}^n-\mathit{\ln}\left({p}_{\mathrm{chosen}}\right)$ and *k* represents the number of parameters in the model.

### Experimental schedule

The experimental schedule is presented in Fig. 1. Initially, rats were trained for the PRL test as described above. After achieving a stable performance resulting in at least 3 performed reversals and less than 15% omissions in three consecutive PRL training sessions, animals that reached the criterion were subsequently tested in 10 consecutive PRL tests over 10 days. On the basis of this ‘sensitivity screening’, the rats were divided in two ways: first, they were divided into the upper and lower quartile of sensitivity to negative feedback and also into the upper and lower quartile of sensitivity to positive feedback. The division according to sensitivity to negative feedback was made based on the average ratio of lever changes following misleading punishment (probabilistic lose-shifts) made by the animals across all 10 screening tests. The division according to the sensitivity to positive feedback was made based on the average ratio of pressing the same lever (win-stays) following both true and misleading rewards across all 10 screening tests. To confirm the stability of the feedback sensitivity traits, we additionally analysed the ‘frequency of sensitivity’, expressed as the number of PRL tests (out of the 10 comprising screening) in which an animal displayed value of sensitivity from the upper quartile of the entire cohort. After the feedback sensitivity screening, the animals were tested in a battery of behavioural tests aimed at measuring behavioural correlates of depression in rodents. Appetitive motivation was assessed in the PRSR paradigm, hedonic status was evaluated in the SP test, locomotor and exploratory activities were measured in the OF test, and finally, the level of anxiety was measured in the LDB test.

Because the rats did not learn the tasks that were used for testing of the differences between the feedback-sensitive and feedback-insensitive animals, the order of tasks was not counterbalanced, e.g. behavioural tests followed feedback screening and not other way around. Conducted tests used to assess the natural propensity of animals to prefer sweet sucrose solution and to avoid brightly lit, open spaces, were unlikely affected by the PRL training procedure. In case of the PRSR testing, the applied order of tests was dictated by the fact that the lever delivering a reward could have become more salient what could affect performance of rats in the PRL paradigm (unless conducted first).

Lastly, to further assess potential differences between the feedback-sensitive and feedback-insensitive animals, three different reinforcement-learning models were fitted (and compared against the ‘random choice’ model) to the behavioural data from the feedback sensitivity screening.

### Statistics

We analysed the data using SPSS (version 25.0, SPSS Inc., Chicago, IL, USA). The normality of the sensitivity to feedback data was verified using the Kolmogorov-Smirnov test. The screening data were analysed using one-way (for the whole cohort) or two-way (for the animals classified as sensitive and insensitive to feedback) ANOVAs with repeated measures and the within-subject factor of test day (10 levels: test day 1 … test day 10) and between-subject factor of feedback sensitivity (2 levels: sensitive and insensitive). The differences between ‘sensitive’ and ‘insensitive’ animals in motivation to gain reward, hedonic status, locomotor activity, exploratory activity and anxiety levels were analysed separately using *t* tests or, for non-parametric data, using Mann-Whitney *U* tests. For pair-wise comparisons, we adjusted the values using Sidak’s correction factor for multiple comparisons (Howell 1997). All of the tests of significance were performed at *α* = 0.05. We tested the homogeneity of variance using Levene’s test, and for repeated-measures analyses, we confirmed sphericity using Mauchly’s test. The data are presented as the mean ± SEM.

## Results

All animals fulfilled the training criteria and qualified for the PRL screening. On average, the animals reached the criteria after 16 ± 1 PRL tests.

### Feedback sensitivity screening

#### Whole cohort

Statistical analysis of the feedback sensitivity screening data revealed that the animals’ performance was stable (non-significant effects of test day on number of reversals (*F*_(9, 351)_ = 1.817, NS), on the proportion of lose-shift behaviours following misleading negative feedback (*F*_(9, 351)_ = 1.132, NS), and on the average proportion of win-stay behaviours following positive (true and misleading) feedback (*F*_(9, 351)_ = 1.651, NS)).

The animals made on average 7.1 ± 0.19 reversals per test session. The average proportion of lose-shift behaviours following misleading negative feedback was 0.52 ± 0.09. The average proportion of win-stay behaviours following true and misleading positive feedback was 0.78 ± 0.01.

The feedback sensitivity screening allowed us to divide animals into groups of those that were permanently more sensitive (top quartile)/and permanently less sensitive (bottom quartile) to the negative or positive feedback.

### Negative feedback sensitivity screening

The average proportion of lose-shift behaviours following misleading negative feedback in animals that was classified as insensitive to negative feedback (bottom quartile of sensitivity) ranged from 0.33 to 0.46 with an average of 0.42 ± 0.01, while in those classified as sensitive (top quartile of sensitivity), the proportion ranged from 0.56 to 0.74 with an average of 0.63 ± 0.02.

The average proportion of win-stay behaviours following true and misleading positive feedback in animals classified as insensitive to negative feedback was 0.79 ± 0.01, while in those classified as sensitive, the average was 0.83 ± 0.02.

**Fig. 2:**
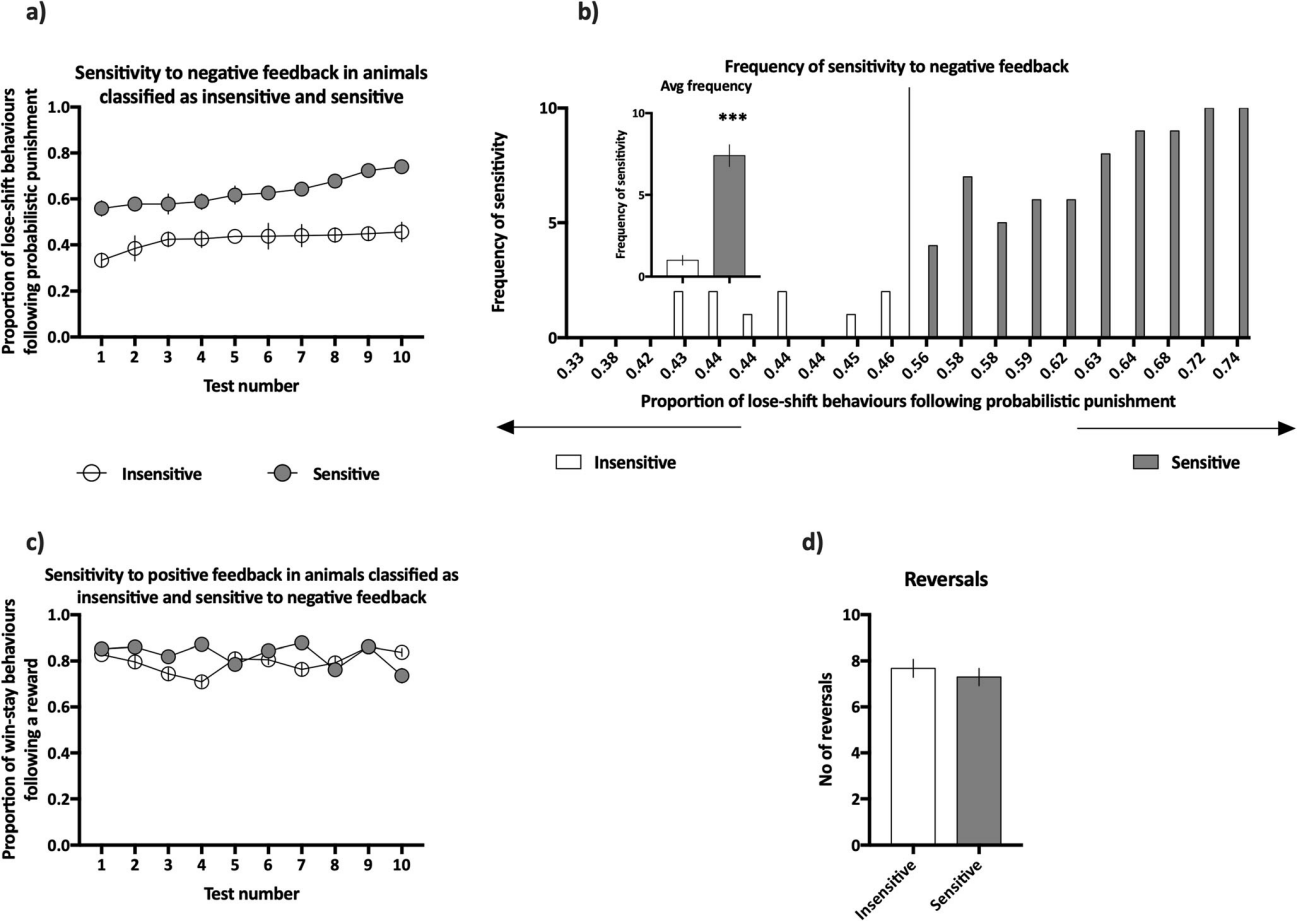
Results of the negative feedback sensitivity screening. **a** Average proportion of lose-shift behaviours following misleading punishment in rats classified as insensitive (open circles, *N* = 10) and sensitive (filled circles, *N* = 10) to negative feedback across all 10 screening probabilistic reversal learning (PRL) tests. **b** Average frequency of sensitivity to negative feedback, expressed as the number of PRL tests (out of the 10 comprising screening) in which an animal displayed value of sensitivity located in the upper quartile of the values from the entire cohort. **b** (Inset) Average frequency of sensitivity to negative feedback in animals classified as insensitive (open bars, *N* = 10) and sensitive to negative (filled bars, *N* = 10) feedback. **c** Average proportion of win-stay behaviours following a reward in rats classified as insensitive (open circles, *N* = 10) and sensitive to negative (filled circles, *N* = 10) feedback across all 10 screening PRL tests. **d** Average number of reversals made by animals classified as insensitive (open bars, *N* = 10) and sensitive to negative (filled bars, *N* = 10) feedback during the 10 screening PRL tests. Data are presented as the mean ± SEM. ***p < 0.001 compared to the insensitive group

Within the top and bottom quartiles, the sensitivity to negative feedback was stable across all 10 screening tests (non-significant effect of test-day (*F*_(9,162)_ = 0.76, NS) and non-significant test-day × feedback sensitivity interaction (*F*_(9,162)_ = 0.48, NS, see Fig. 2a)). The groups of animals classified as sensitive and insensitive to negative feedback did not significantly differ in their sensitivity to positive feedback (non-significant effect of feedback sensitivity, *F*_(1,18)_ = 2.32, NS, see Fig. 2c) or in the numbers of reversals made during the screening (non-significant effect of feedback sensitivity, *F*_(1,18)_ = 0.48, NS, see Fig. 2d).

The average frequency of sensitivity in the group of animals classified as sensitive to negative feedback was significantly (*p* > 0.001) higher than that in the group classified as insensitive to negative feedback ((*t*_(18)_ = 8.727), see Fig. 2b and b inset).

### Positive feedback sensitivity screening

The average proportion of win-stay behaviours following true and misleading positive feedback in animals classified as insensitive to positive feedback (bottom quartile of sensitivity) ranged from 0.66 to 0.74 with an average of 0.70 ± 0.01, while in those classified as sensitive to positive feedback (upper top quartile of sensitivity), the proportion ranged from 0.84 to 0.88 with an average of 0.86 ± 0.02.

The average proportion of lose-shift behaviours following misleading negative feedback in animals classified as insensitive to positive feedback was 0.53 ± 0.03, while in those classified as sensitive, the average was 0.56 ± 0.03.

**Fig. 3:**
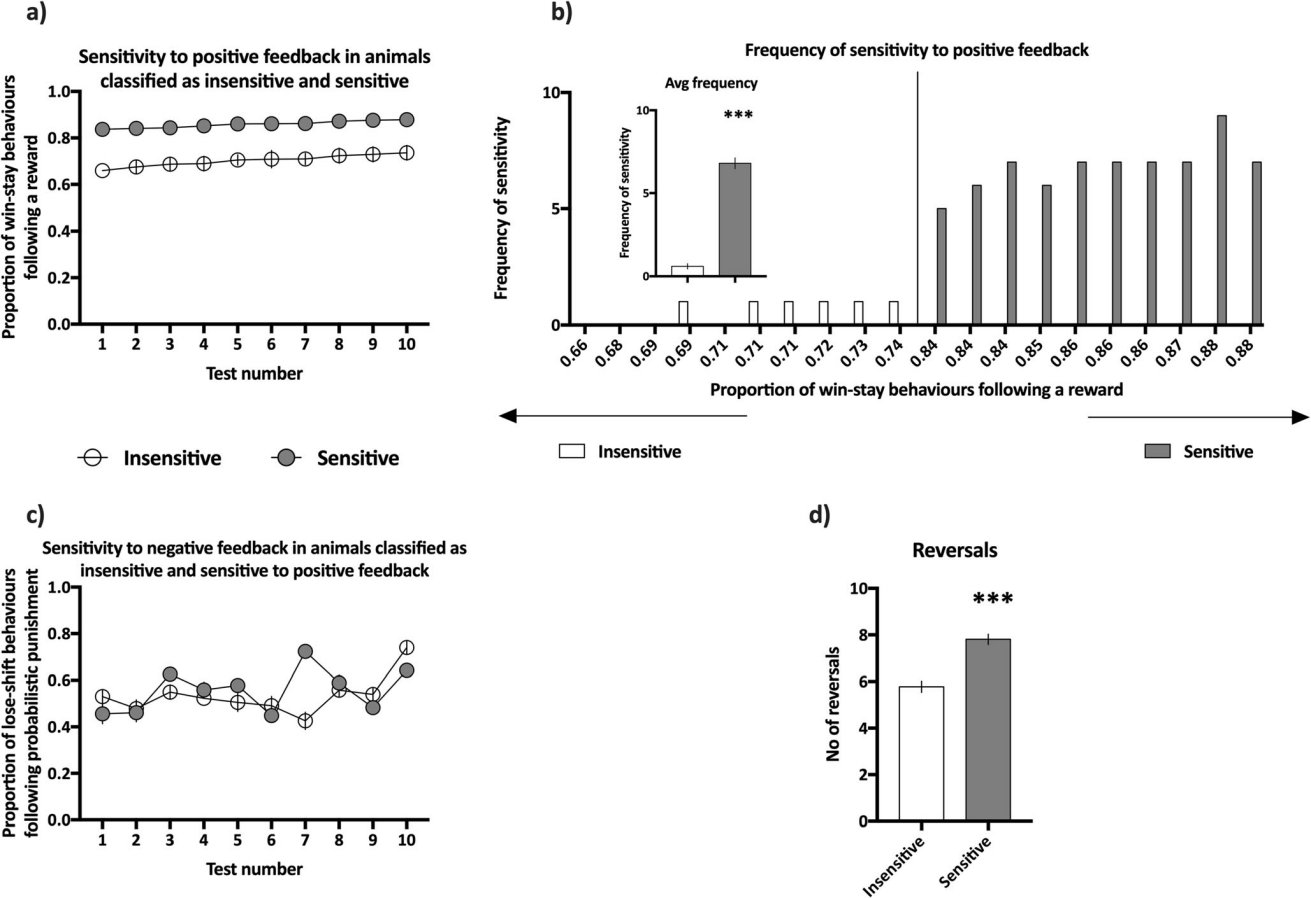
Results of the positive feedback sensitivity screening. **a** Average proportion of win-stay behaviours following a reward in rats classified as insensitive (open circles, *N* = 10) and sensitive (filled circles, *N* = 10) to positive feedback across all 10 screening probabilistic reversal learning (PRL) tests. **b** Average frequency of sensitivity to positive feedback, expressed as the number of PRL tests (out of the 10 comprising screening) in which an animal displayed value of sensitivity located in the upper quartile of the values from the entire cohort. **b** (Inset) Average frequency of sensitivity to positive feedback in animals classified as insensitive (open bars, *N* = 10) and sensitive to positive feedback (filled bars, *N* = 10). **c** Average proportion of lose-shift behaviours following a reward in rats classified as insensitive (open circles, *N* = 10) and sensitive (filled circles, *N* = 10) to positive feedback across all 10 screening PRL tests. **d** Average number of reversals made by animals classified as insensitive (open bars, *N* = 10) and sensitive (filled bars, *N* = 10) to positive feedback during the 10 screening PRL tests. Data are presented as the mean ± SEM. ***p < 0.001 compared to the insensitive group

Within the upper and lower quartiles, the sensitivity to positive feedback was stable across all 10 screening tests (non-significant effect of test-day (*F*_(9,162)_ = 1.37, NS) and non-significant test-day × feedback sensitivity interaction (*F*_(9,162)_ = 0.62, NS, see Fig. 3a)). The groups of animals classified as sensitive and insensitive to positive feedback did not significantly differ in their sensitivity to negative feedback (non-significant effect of feedback sensitivity, *F*_(1,18)_ = 0.33, NS, see Fig. 3c). The animals classified as sensitive to positive feedback made on average significantly (*p* < 0.001) more reversals during the screening tests than those that were classified as insensitive to positive feedback did (significant effect of feedback sensitivity, *F*_(1,18)_ = 38.14, see Fig. 3d).

The average frequency of sensitivity in the group of animals classified as sensitive to positive feedback was significantly (*p* > 0.001) higher than that in the group classified as insensitive to positive feedback (*t*_(18)_ = 16.37, see Fig. 3b and b inset).

### General

Following feedback sensitivity screening, 30 out of 40 experimental animals (belonging to the upper and lower quartiles of sensitivity to positive and negative feedback) have undergone further behavioural tests and analyses. Out of these, 6 rats were sensitive to both types of feedback, 2 were sensitive to positive feedback and insensitive to negative feedback, 1 was sensitive to negative feedback and insensitive to positive feedback, 2 were exclusively sensitive to positive feedback, 3 were exclusively sensitive to negative feedback, 8 were exclusively insensitive to positive feedback, 7 were exclusively insensitive to negative feedback, and only 1 rat was insensitive to both types of feedback.

The difference in the proportion of lose-shift behaviours between rats classified as sensitive and insensitive to negative feedback (0.21 ± 0.003) was statistically significantly (*p* ≤ 0.01) higher than the difference in the proportion of win-stay behaviours between animals classified according to positive feedback sensitivity (0.16 ± 0.014, *t*_(18)_ = 3.741).

#### Effects of negative feedback sensitivity on cognitive, affective, and motivational correlates of depression in rats

**Fig. 4:**
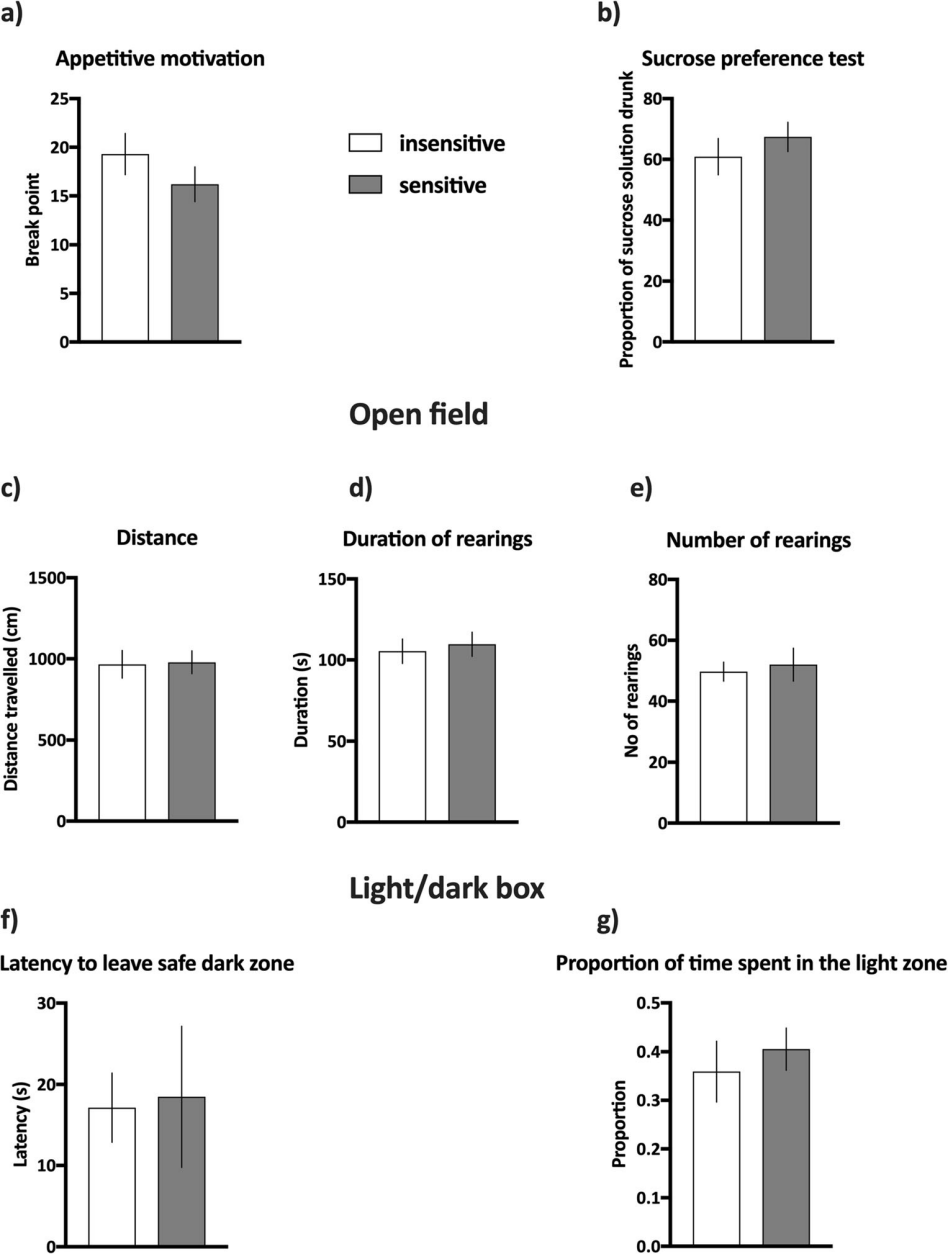
Effects of trait sensitivity to negative feedback on motivational, hedonic and affective correlates of depressive symptoms in rats. **a** Appetitive motivation of animals classified as insensitive (open bars) and sensitive (filled bars) to negative feedback expressed as a break point in the progressive ratio schedule of reinforcement (PRSR) paradigm. **b** Hedonic status expressed as preference to sweet sucrose solution in the sucrose preference (SP) test. **c** Horizontal activity expressed by distance travelled in the open field (OF) test. **d**, **e** Vertical activities in the OF test expressed as the duration and number of rearings, respectively. **f**, **g** The level of anxiety measured in the LDB test and expressed by the latency to leave the safe/dark zone and proportion of time spent in the light zone, respectively. Data are presented as the mean ± SEM. *N* = 10 rats per group

In the PRSR paradigm, the rats classified as insensitive and sensitive to negative feedback stopped responding when the average number of lever presses necessary to obtain a reward reached an average of 19.3 ± 2.1 and 16.2 ± 1.8, respectively (see Fig. 4a). Sensitivity to negative feedback had no statistically significant effects on the average breaking point of rats in the PRSR test (Mann-Whitney *U* = 36, *p* = 0.028, NS, see Fig. 4a).

We also observed no statistically significant differences in the sucrose preference between groups of animals that were sensitive and insensitive to negative feedback (*t*_(18)_ = 0.85, NS, Fig. 4b). The rats classified as insensitive and sensitive to negative feedback showed an average of 61 ± 6% and 67 ± 5% sucrose preferences, respectively (see Fig. 4b).

Similarly, the results from the OF tests revealed no statistically significant differences between groups of animals that were sensitive or insensitive to negative feedback. The experimental groups did not differ in the intensity of horizontal exploration (distance travelled by insensitive animals 966.2 cm ± 86.1, by sensitive animals 978.6 cm ± 70.31 (*t*_(18)_ = 0.11, NS, Fig. 4c)), in the time (insensitive animals 105.4 s ± 7.6, sensitive animals 109.7 s ± 77.5 (Mann-Whitney *U* = 45, *p* = 0.74, NS, see Fig. 4d)) or in the number of rearings (insensitive animals 49.7 ± 3.1, sensitive animals 52.0 ± 5.5 (*t*_(18)_ = 0.37, NS, see Fig. 4e)).

Finally, the results from the LDB test also revealed no statistically significant interactions between sensitivity to negative feedback and behavioural correlates of anxiety in rodents. The latency to leave a safe dark box did not significantly differ between experimental groups (insensitive animals 17.1 s ± 4.3, sensitive animals 18.49 s ± 8.7 (Mann-Whitney *U* = 39, *p* = 0.42, NS, see Fig. 4f)). We also observed no statistically significant differences in the proportion of time spent in the light zone (insensitive animals 0.36 ± 0.06, sensitive animals 0.41 ± 0.04 (*t*_(18)_ = 0.60, NS, see Fig. 4g)).

#### Effects of positive feedback sensitivity on cognitive, affective and motivational correlates of depression in rats

**Fig. 5:**
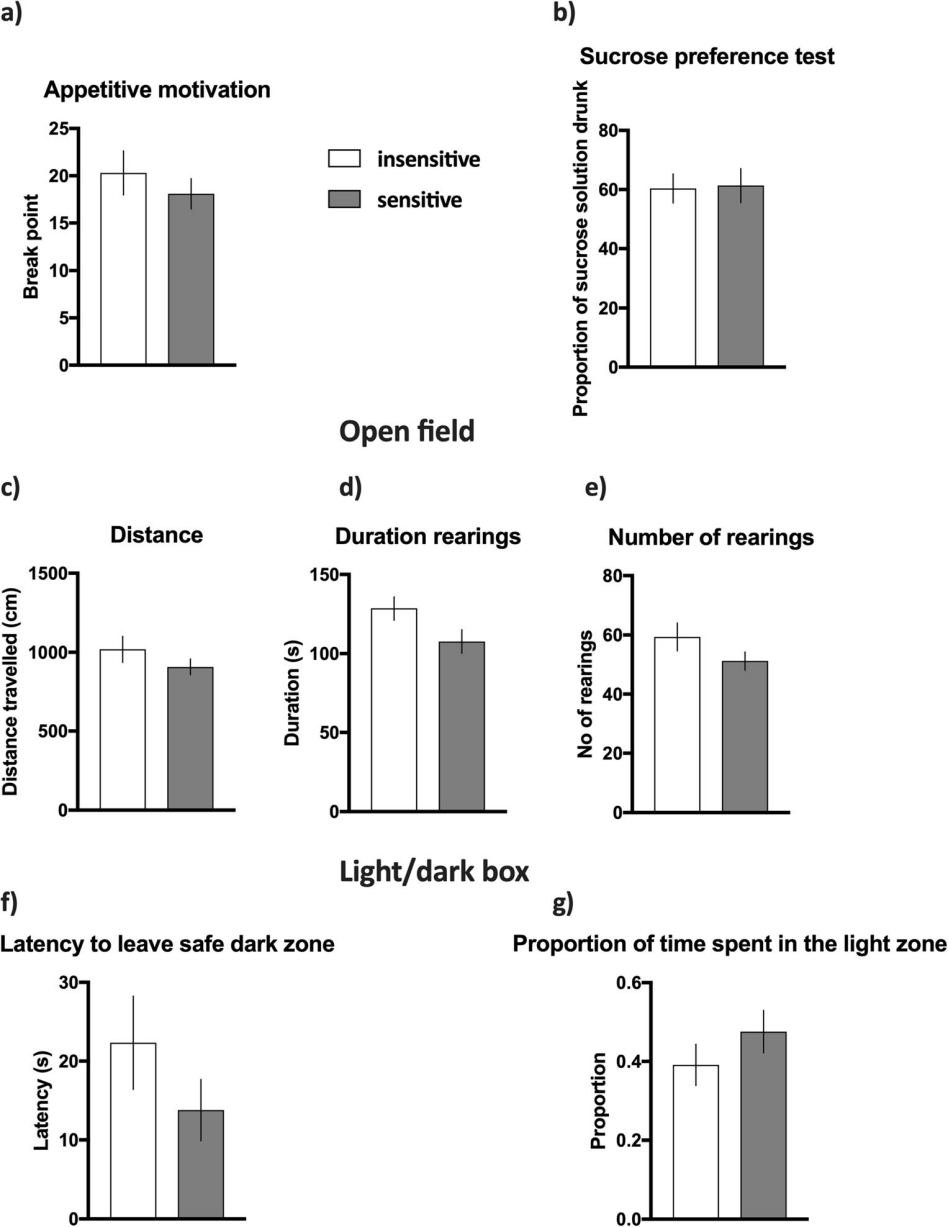
Effects of trait sensitivity to positive feedback on motivational, hedonic and affective correlates of depressive symptoms in rats. **a** Appetitive motivation of animals classified as insensitive (open bars) and sensitive (filled bars) to positive feedback expressed as a break point in the progressive ratio schedule of reinforcement (PRSR) paradigm. **b** Hedonic status expressed as preference to sweet sucrose solution in the sucrose preference (SP) test. **c** Horizontal activity expressed by distance travelled in the open field (OF) test. **d**, **e** Vertical activities in the OF test expressed as the duration and number of rearings, respectively. **f**, **g** The level of anxiety measured in the LDB test and expressed by the latency to leave the safe/dark zone and proportion of time spent in the light zone, respectively. Data are presented as the mean ± SEM. *N* = 10 rats per group

The rats classified as insensitive and sensitive to positive feedback stopped responding when the average number of lever presses necessary to obtain a reward reached an average of 20.3 ± 2.3 and 18.1 ± 1.6, respectively (see Fig. 5a). Sensitivity to positive feedback had no statistically significant effects on the average breaking point of rats in the PRSR test (*t*_(18)_ = 0.78, NS, see Fig. 5a).

We also observed no statistically significant differences in the sucrose preference between groups of animals that were sensitive and insensitive to positive feedback (*t*_(18)_ = 0.13, NS, see Fig. 5b). The rats classified as insensitive and sensitive to positive feedback showed an average of 60 ± 5% and 61 ± 6% of sucrose preference, respectively (Fig. 5b).

Similarly, the results from OF tests revealed no statistically significant differences between groups of animals that were sensitive and insensitive to positive feedback. The experimental groups did not differ in the intensity of horizontal exploration (distance travelled by insensitive animals 1019.0 cm ± 83.1, by sensitive animals 907.4 cm ± 50.3 (*t*_(18)_ = 1.15, NS), see Fig. 5c), in the time (insensitive animals 128.5 s ± 7.3, sensitive animals 107.6 s ± 7.5 (*t*_(18)_ = 1.99, NS, see Fig. 5d)) or in number of rearings (insensitive animals 59.3 ± 4.8, sensitive animals 51.2 ± 3.1 (Mann-Whitney *U* = 36, *p* = 0.31, NS, see Fig. 5e)).

Finally, the results from the LDB test also revealed no statistically significant interactions between sensitivity to positive feedback and behavioural correlates of anxiety in rodents. The latency to leave a safe dark box did not significantly differ between experimental groups (insensitive animals 22.3 s ± 5.9, sensitive animals 13.8 s ± 3.9 (*t*_(18)_ = 1.21, NS, see Fig. 5f)). We also observed no statistically significant differences in the proportion of time spent in the light zone (insensitive animals 0.39 ± 0.05, sensitive animals 0.48 ± 0.05 (*t*(18) = 1.12, NS, see Fig. 5g)).

#### Computational modelling

**Fig. 6:**
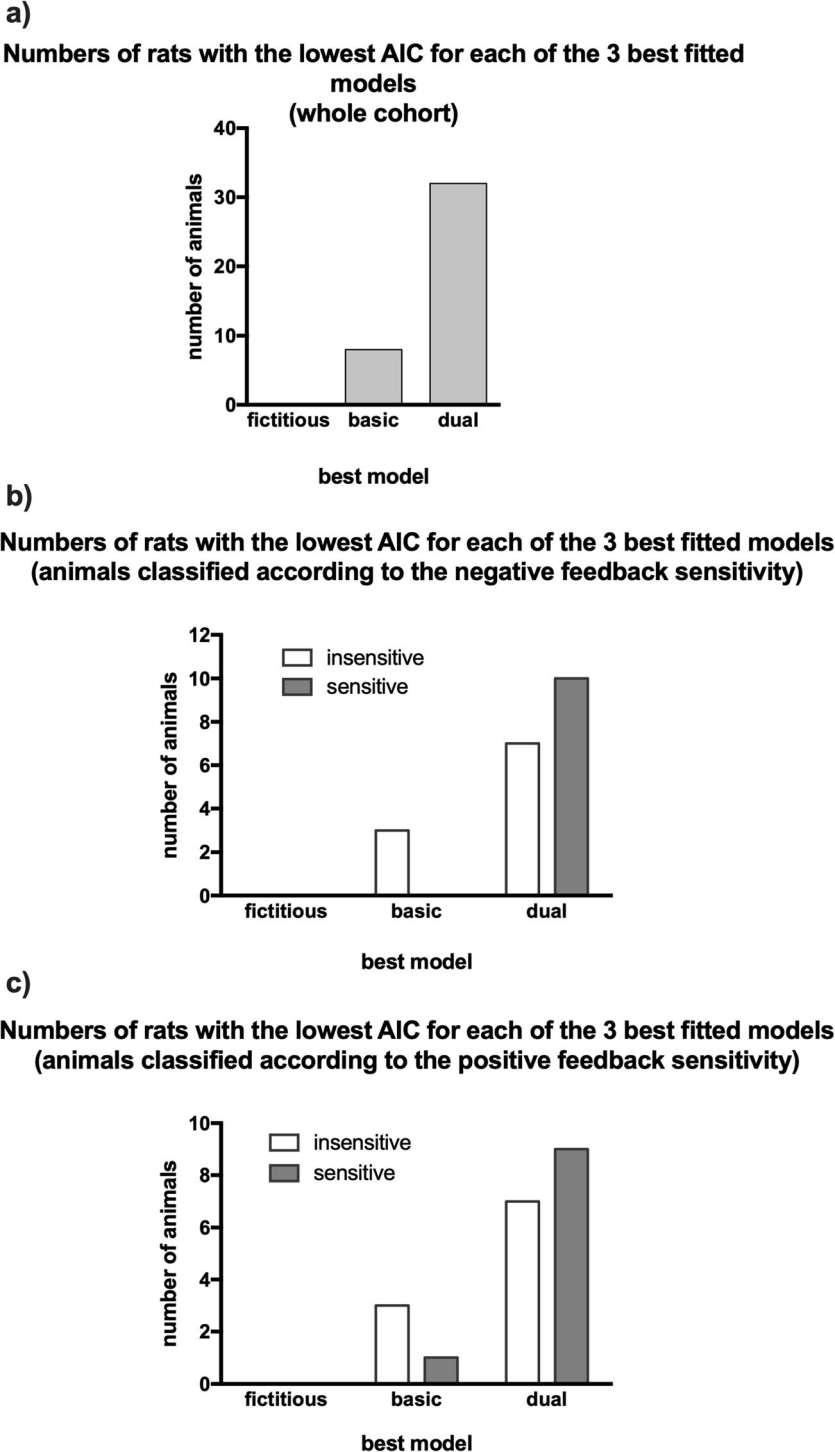
Numbers with the lowest AIC for each of the 3 best-fit models. Graphs show the numbers of rats with the lowest AICs for each of the 3 best-fit models for **a** the total cohort of animals, **b** the animals classified as sensitive and insensitive to negative feedback and **c** the animals classified as sensitive and insensitive to positive feedback

To further assess potential differences between the feedback sensitive and insensitive groups, we fitted reinforcement learning models to the observed choices. Three models were considered and analysed along with the fourth, the ‘random choice’ model, which assumed that the choices were random. The simplest, ‘basic’ model assumed a single learning rate for both positive and negative choice outcomes. The second model, ‘dual’, introduced separate learning rates, while the third, ‘fictitious’, assumed a single learning rate but updated an expected value for the non-selected option. In all cases, the probabilities of specific choices were calculated using a softmax policy, with the inverse temperature β parameter determining the effect of the difference between expected values on choice. Additionally, we applied a random choice model as a control (i.e., 0.5 probability of selecting either option at each step). The model parameters were optimized to achieve the lowest sum of the nll of actually selected choices at each step. Comparison of model fits showed that the ‘dual’ model yielded the lowest AIC scores in 32, the ‘basic’ model did in 8, while ‘fictitious’ yielded the lowest AIC score in none out of the 40 analysed rats (Fig. 6a). For all three analysed models, the AIC scores were lower than that for the ‘random choice’ model (see Table 1).

**Table 1:** Model comparisons using AIC. The AIC, α and β values for each model are presented as the mean ± SEM. The best-fit model is italicized

	‘Basic’	‘Dual’	‘Fictitious’	‘Random choice’
AIC (± SEM)	2122.96 (29.14)	*2115.89 (29.43)*	2571.74 (17.63)	2719.81 (14.82)
α (± SEM)	0.68579 (0.01982)	*0.56771* ^a^ *(0.02418)*	0.47635 (0.03135)	n/a
		*0.76578* ^b^ *(0.02197)*		
β (± SEM)	1.90044 (0.04916)	*2.03208 (0.05885)*	3.43467 (1.251)	n/a

*n/a* not applicable

^a^α+

^b^α−

Based on the sensitivity to negative feedback, the comparison of model fits showed that ‘dual’ yielded the lowest AIC scores in 7 insensitive and 10 sensitive animals, while the ‘basic’ did in 3 insensitive and 0 sensitive animals out of 20 (bottom (*n* = 10) and top (*n* = 10) quartiles of sensitivity) rats (Fig. 6b).

Based on the sensitivity to positive feedback, the comparison of model fits showed that ‘dual’ yielded the lowest AIC scores in 7 insensitive and 9 sensitive animals, while the ‘basic’ did in 3 insensitive and 1 sensitive animals out of 20 (bottom (*n* = 10) and top (*n* = 10) quartiles of sensitivity) rats (Fig. 6c).

**Fig. 7:**
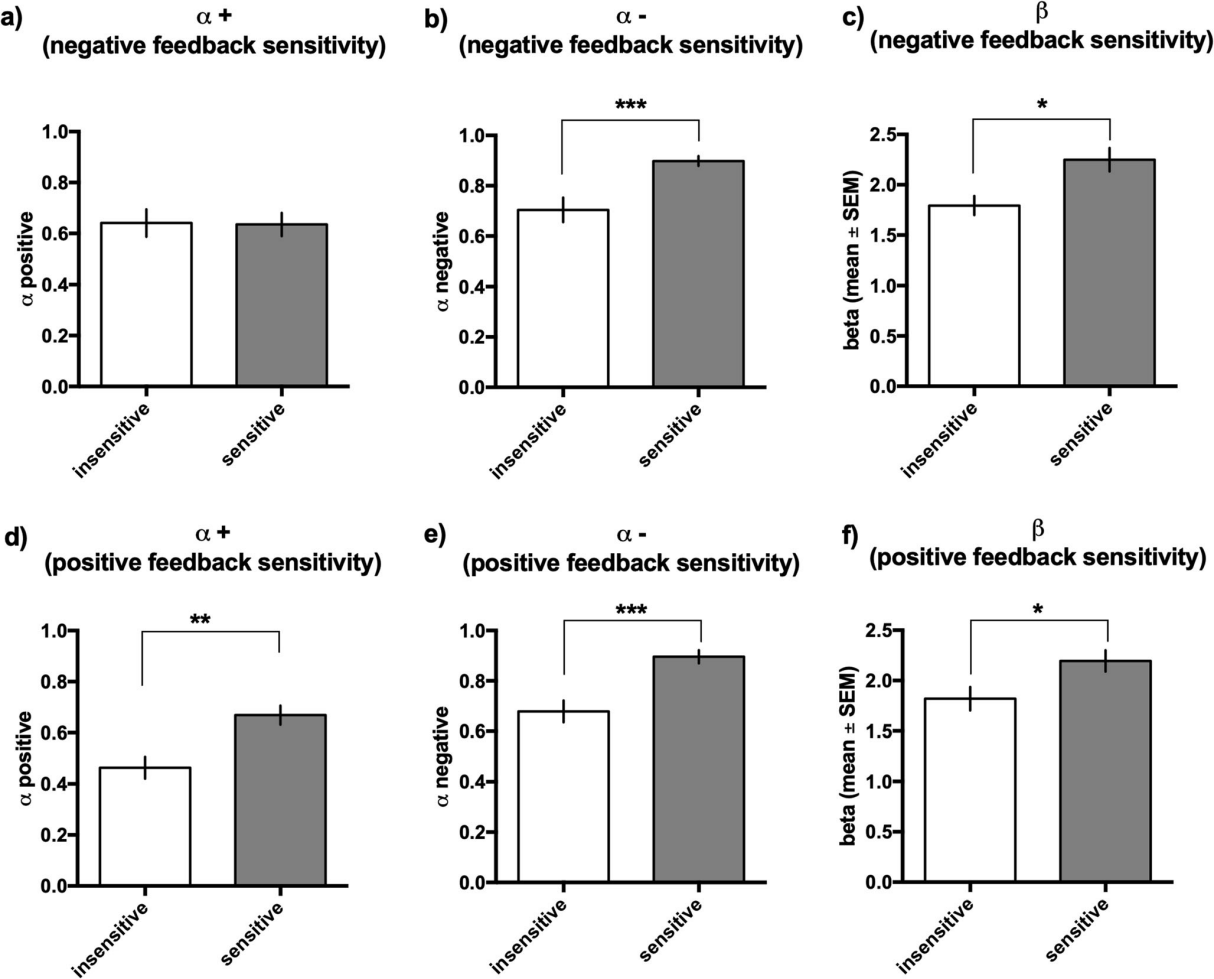
Parameters for the ‘dual’ model in rats classified according to negative and positive feedback sensitivity. Differences between animals classified as sensitive and insensitive to negative (**a**–**c**) and positive (**d**–**f**) feedback in α+ (**a**, **d**), α− (**b**, **e**) and β (**c**, **f**) parameters in the ‘dual’ model. The data are presented as the mean ± SEM (*n* = 10). *p < 0.05, **p < 0.01, ***p < 0.001

We found that the optimal model parameters corresponding to animals in the top and bottom quartiles of sensitivity to negative or positive feedback differ significantly (see Fig. 7). The animals from the top quartile of negative feedback sensitivity (Fig. 7a, b and c) had significantly higher α− rates (as intuitively expected) and significantly higher β values than the animals from the bottom quartile of sensitivity did (*p* < 0.001, 0.05, respectively). Interestingly, the animals from the top quartile of positive feedback sensitivity (Fig. 7d, e and f) had significantly higher α+ rates (as intuitively expected), significantly higher α− rates and significantly higher β values than the animals from the bottom quartile of sensitivity did (*p* < 0.01, 0.001, 0.05, respectively).

## Discussion

The results of our study demonstrate that in rodents, sensitivity to negative and positive feedback could be considered stable and enduring behavioural traits. Importantly, we also showed that these traits are independent of each other and that trait sensitivity to positive feedback is associated with cognitive flexibility in the PRL test. The results of computational modelling also revealed that in animals classified as sensitive to positive feedback, the α learning rates for both positive and negative reward prediction errors were higher than the α learning rates in those classified as insensitive. We observed no statistically significant interactions between sensitivity to negative or positive feedback and parameters measured in the PRSR, SP, OF or LDB tests.

Empirical findings in many non-human species, ranging from insects to mammals, suggest that personality traits are a widespread phenomenon in the animal kingdom (Gosling 2001). Animals differ profoundly from each other in their behaviour, and these differences are often consistent over time and extend to various contexts. A growing body of experimental evidence also suggests that laboratory animals may be used to investigate interactions between various personality traits and vulnerability to psychiatric disorders. For instance, in recent studies from our laboratory, we demonstrated that in rats, trait ‘pessimism’ is associated with a ‘pro-depressive profile’ that predicts increased vulnerability to stress-induced motivational deficits (Drozd et al. 2017) and stress-induced anhedonia (Rygula et al. 2013), both of which are important symptoms of depression in humans (DSM-5 2013).

The results of our present study revealed that another information processing bias that is associated with depressive disorder in humans, high/low sensitivity to performance feedback could be considered a behavioural trait in laboratory animals. Analysis of the frequency of sensitivity to the negative and positive feedback across 10 tests comprising the screening period revealed that these traits are not only qualitative, as indexed by the ratio of lose-shift and win-stay behaviours, respectively, but are also quantitative. The animals classified as sensitive to negative feedback displayed high sensitivity (top quartile of lose-shift ratios) significantly more frequently than their insensitive conspecifics did, indicating stability and endurance of the trait. The same was true for animals classified according to their sensitivity to positive feedback.

Interestingly, although the logic behind sensitivity to feedback construct could assume a bipolar dimension, with a high sensitivity to positive feedback and a low sensitivity to negative feedback at one end and the vice versa at the other, with a neutral point in the middle, these traits seem to be completely separable and independent of each other and have different consequences. The trait sensitivity to positive feedback was associated with a better PRL performance as indexed by a statistically higher number of reversals made by the animals sensitive to positive feedback than the number made by those that were less sensitive. This better performance likely resulted from the statistically significantly higher rates of learning from both positive and negative reward prediction errors (α+ and α−, respectively) in animals classified as sensitive to positive feedback in comparison to their insensitive conspecifics. Moreover, in both cases, fitted β values were higher in sensitive animals, indicating that they were more likely to select the choice with higher expected value.

There are several limitations in this study that need to be mentioned. When considered in the context of depression, the first limitation would be the use of only male subjects. Indeed, the prevalence of depressive disorder is significantly higher in women than in men. However, the decision of using only male subjects was based on practical reasons: males do not have oestrous cycle that could quite likely, by itself, affect the sensitivity to feedback. Since the studies investigating feedback sensitivity in the preclinical models of probabilistic reversal learning are still pioneering (the landmark study by Bari et al., was published only 9 years ago) we decided to avoid any additional confounding factors that could affect our measures and tested only male rats.

Secondly, the higher number of reversals made by the animals that were more sensitive to positive feedback could account not for the higher cognitive flexibility but result from the nature of the PRL task, where the experimental ‘sticks’ were less salient (being just lack of reward) than the experimental ‘carrots’. However, the results from PRSR and SP tests, demonstrated clearly no significant differences in motivation to gain a reward and in the reward sensitivity between animals classified as sensitive and insensitive to positive feedback. The abovementioned observation suggests something more than just the reward salience that contributes to the observed effects. Further studies should help to disentangle processes that constitute feedback sensitivity in this pre-clinical PRL task.

Finally, it might be interesting to analyse the data in a model that includes both types of feedback sensitivity instead of looking at sensitivity to the positive and negative feedback separately. This natural next step would imply an analysis where one could separate of several interesting behavioural phenotypes, i.e. hypersensitive to negative feedback and insensitive to positive feedback (‘pro-depressive’) or vice versa (‘pro-manic’). Such analysis however would require using more experimental animals.

In our study, we observed no statistically significant effects of trait sensitivity to feedback on cognitive, hedonic or affective correlates of depressive symptoms in rodents measured in the PRSR, SP, OF and LDB paradigms. The rats showed fair performance in the PRSR paradigm, indicating normal levels of motivation to obtain a reward as well as modest but apparent preference for the 0.8% sucrose solution in the SP test, demonstrating normal levels of reward sensitivity. They also showed normal levels of exploration in the OF test and significant preference for the dark zone of the LDB. These results demonstrate that in healthy rats, increased/decreased sensitivity to negative/positive feedback does not interact with other domains of processes that are usually altered in depression. This result, however, is not surprising. Although according to Beck’s cognitive model of depression, biased acquisition and processing of information, including increased/decreased sensitivity to negative/positive feedback, has a primary role in the development and maintenance of depression (Beck 1987, 2008), the development of depressive symptoms usually requires environmental triggers, e.g. stress (Beck 1987, 2008). The same could be true in rats. Further studies using animal models of depression based on chronic stress should reveal whether sensitivity to feedback is a latent trait that when interacts with stressful life events, could produce correlates of depressive symptoms in rats.

## References

[CR1] Akaike H (1981) Citation classic - a new look at the statistical-model identification. Cc/Eng Tech Appl Sci 22–22

[CR2] Bari A, Theobald DE, Caprioli D, Mar AC, Aidoo-Micah A, Dalley JW, Robbins TW (2010). Serotonin modulates sensitivity to reward and negative feedback in a probabilistic reversal learning task in rats. Neuropsychopharmacology.

[CR3] Beats BC, Sahakian BJ, Levy R (1996). Cognitive performance in tests sensitive to frontal lobe dysfunction in the elderly depressed. Psychol Med.

[CR4] Beck AT (1967). Depression: clinical, experimental, and theoretical aspects.

[CR5] Beck AT (1987). Cognitive models of depression. J Cogn Psychother.

[CR6] Beck AT (2008). The evolution of the cognitive model of depression and its neurobiological correlates. Am J Psychiatry.

[CR7] Brittlebank AD, Scott J, Williams JM, Ferrier IN (1993). Autobiographical memory in depression: state or trait marker?. Br J Psychiatry.

[CR8] Dalton GL, Phillips AG, Floresco SB (2014). Preferential involvement by nucleus accumbens shell in mediating probabilistic learning and reversal shifts. J Neurosci.

[CR9] Dalton GL, Wang NY, Phillips AG, Floresco SB (2016). Multifaceted contributions by different regions of the orbitofrontal and medial prefrontal cortex to probabilistic reversal learning. J Neurosci.

[CR10] den Ouden HE, Daw ND, Fernandez G, Elshout JA, Rijpkema M, Hoogman M, Franke B, Cools R (2013). Dissociable effects of dopamine and serotonin on reversal learning. Neuron.

[CR11] Disner SG, Beevers CG, Haigh EA, Beck AT (2011). Neural mechanisms of the cognitive model of depression. Nat Rev Neurosci.

[CR12] Drozd R, Rojek-Sito K, Rygula R (2017). The trait ‘pessimism’ does not interact with cognitive flexibility but makes rats more vulnerable to stress-induced motivational deficits: results from the attentional set-shifting task. Behav Brain Res.

[CR13] Drozd R, Rychlik M, Fijalkowska A, Rygula R (2019). Effects of cognitive judgement bias and acute antidepressant treatment on sensitivity to feedback and cognitive flexibility in the rat version of the probabilistic reversal-learning test. Behav Brain Res.

[CR14] DSM-5 (2013). Diagnostic and statistical manual of mental disorders.

[CR15] Elliott R, Sahakian BJ, Herrod JJ, Robbins TW, Paykel ES (1997). Abnormal response to negative feedback in unipolar depression: evidence for a diagnosis specific impairment. J Neurol Neurosurg Psychiatry.

[CR16] Glascher J, Hampton AN, O'Doherty JP (2009). Determining a role for ventromedial prefrontal cortex in encoding action-based value signals during reward-related decision making. Cereb Cortex.

[CR17] Gosling SD (2001). From mice to men: what can we learn about personality from animal research?. Psychol Bull.

[CR18] Gotlib IH, Joormann J (2010). Cognition and depression: current status and future directions. Annu Rev Clin Psychol.

[CR19] Grospe GM, Baker PM, Ragozzino ME (2018). Cognitive flexibility deficits following 6-OHDA lesions of the rat dorsomedial striatum. Neuroscience.

[CR20] Howell DC (1997). Statistical methods for psychology.

[CR21] Ineichen C, Sigrist H, Spinelli S, Lesch KP, Sautter E, Seifritz E, Pryce CR (2012). Establishing a probabilistic reversal learning test in mice: evidence for the processes mediating reward-stay and punishment-shift behaviour and for their modulation by serotonin. Neuropharmacology.

[CR22] Maia TV, Frank MJ (2011). From reinforcement learning models to psychiatric and neurological disorders. Nat Neurosci.

[CR23] Murphy FC, Michael A, Robbins TW, Sahakian BJ (2003). Neuropsychological impairment in patients with major depressive disorder: the effects of feedback on task performance. Psychol Med.

[CR24] Nelder JA, Mead R (1965) A simplex method for function minimization. Comput. J 7:308–313

[CR25] Nilsson SR, Alsio J, Somerville EM, Clifton PG (2015). The rat’s not for turning: dissociating the psychological components of cognitive inflexibility. Neurosci Biobehav Rev.

[CR26] Papp M, Willner P, Muscat R (1991). An animal model of anhedonia: attenuation of sucrose consumption and place preference conditioning by chronic unpredictable mild stress. Psychopharmacology.

[CR27] Rescorla RA, Wagner AR (1972) A theory of Pavlovian conditioning: variations in the effectiveness of reinforcement and nonreinforcement, Classical Conditioning II. Appleton-Century-Crofts

[CR28] Roberts DC, Bennett SA (1993). Heroin self-administration in rats under a progressive ratio schedule of reinforcement. Psychopharmacology.

[CR29] Rychlik M, Bollen E, Rygula R (2017). Ketamine decreases sensitivity of male rats to misleading negative feedback in a probabilistic reversal-learning task. Psychopharmacology.

[CR30] Rygula R, Popik P (2016). Trait “pessimism” is associated with increased sensitivity to negative feedback in rats. Cogn Affect Behav Neurosci.

[CR31] Rygula R, Papciak J, Popik P (2013). Trait pessimism predicts vulnerability to stress-induced anhedonia in rats. Neuropsychopharmacology.

[CR32] Rygula R, Golebiowska J, Kregiel J, Kubik J, Popik P (2015). Effects of optimism on motivation in rats. Front Behav Neurosci.

[CR33] Rygula R, Noworyta-Sokolowska K, Drozd R, Kozub A (2018). Using rodents to model abnormal sensitivity to feedback in depression. Neurosci Biobehav Rev.

[CR34] Taylor Tavares JV, Clark L, Furey ML, Williams GB, Sahakian BJ, Drevets WC (2008). Neural basis of abnormal response to negative feedback in unmedicated mood disorders. Neuroimage.

[CR35] Willner P, Muscat R, Papp M (1992). An animal model of anhedonia. Clin Neuropharmacol.

